# Multifractality in stride-to-stride variations reveals that walking involves more movement tuning and adjusting than running

**DOI:** 10.3389/fnetp.2023.1294545

**Published:** 2023-10-19

**Authors:** Taylor J. Wilson, Madhur Mangalam, Nick Stergiou, Aaron D. Likens

**Affiliations:** ^1^ Division of Biomechanics and Research Development, Department of Biomechanics, Center for Research in Human Movement Variability, University of Nebraska at Omaha, Omaha, NE, United States; ^2^ Department of Physical Education and Sport Science, Aristotle University, Thessaloniki, Greece

**Keywords:** complexity, DFA, gait, locomotion, multiscale, multifractal spectrum

## Abstract

**Introduction:** The seemingly periodic human gait exhibits stride-to-stride variations as it adapts to the changing task constraints. The optimal movement variability hypothesis (OMVH) states that healthy stride-to-stride variations exhibit “fractality”—a specific temporal structure in consecutive strides that are ordered, stable but also variable, and adaptable. Previous research has primarily focused on a single fractality measure, “monofractality.” However, this measure can vary across time; strideto-stride variations can show “multifractality.” Greater multifractality in stride-tostride variations would highlight the ability to tune and adjust movements more.

**Methods:** We investigated monofractality and multifractality in a cohort of eight healthy adults during self-paced walking and running trials, both on a treadmill and overground. Footfall data were collected through force-sensitive sensors positioned on their heels and feet. We examined the effects of self-paced walking vs. running and treadmill vs. overground locomotion on the measure of monofractality, α-DFA, in addition to the multifractal spectrum width, W, and the asymmetry in the multifractal spectrum, W*_Asym_*, of stride interval time series.

**Results:** While the α-DFA was larger than 0.50 for almost all conditions, α-DFA was higher in running and locomoting overground than walking and locomoting on a treadmill. Similarly, W was greater while locomoting overground than on a treadmill, but an opposite trend indicated that W was greater in walking than running. Larger W*_Asym_* values in the negative direction suggest that walking exhibits more variation in the persistence of shorter stride intervals than running. However, the ability to tune and adjust movements does not differ between treadmill and overground, although both exhibit more variation in the persistence of shorter stride intervals.

**Discussion:** Hence, greater heterogeneity in shorter than longer stride intervals contributed to greater multifractality in walking compared to running, indicated by larger negative W*_Asym_* values. Our results highlight the need to incorporate multifractal methods to test the predictions of the OMVH.

## Highlights

• Multifractality offers a compelling analytical method to describe how correlations across consecutive stride intervals during locomotion vary across time.
• Stride-to-stride variations show greater multifractality during walking compared to running and locomoting overground compared to a treadmill.
• Greater multifractality reveals that walking involves more movement tuning and adjusting than running.
• Greater multifractality in stride-to-stride variations reveals that locomoting overground allows more freedom to tune and adjust movements than on a treadmill.

## 1 Introduction

**FIGURE 1:**
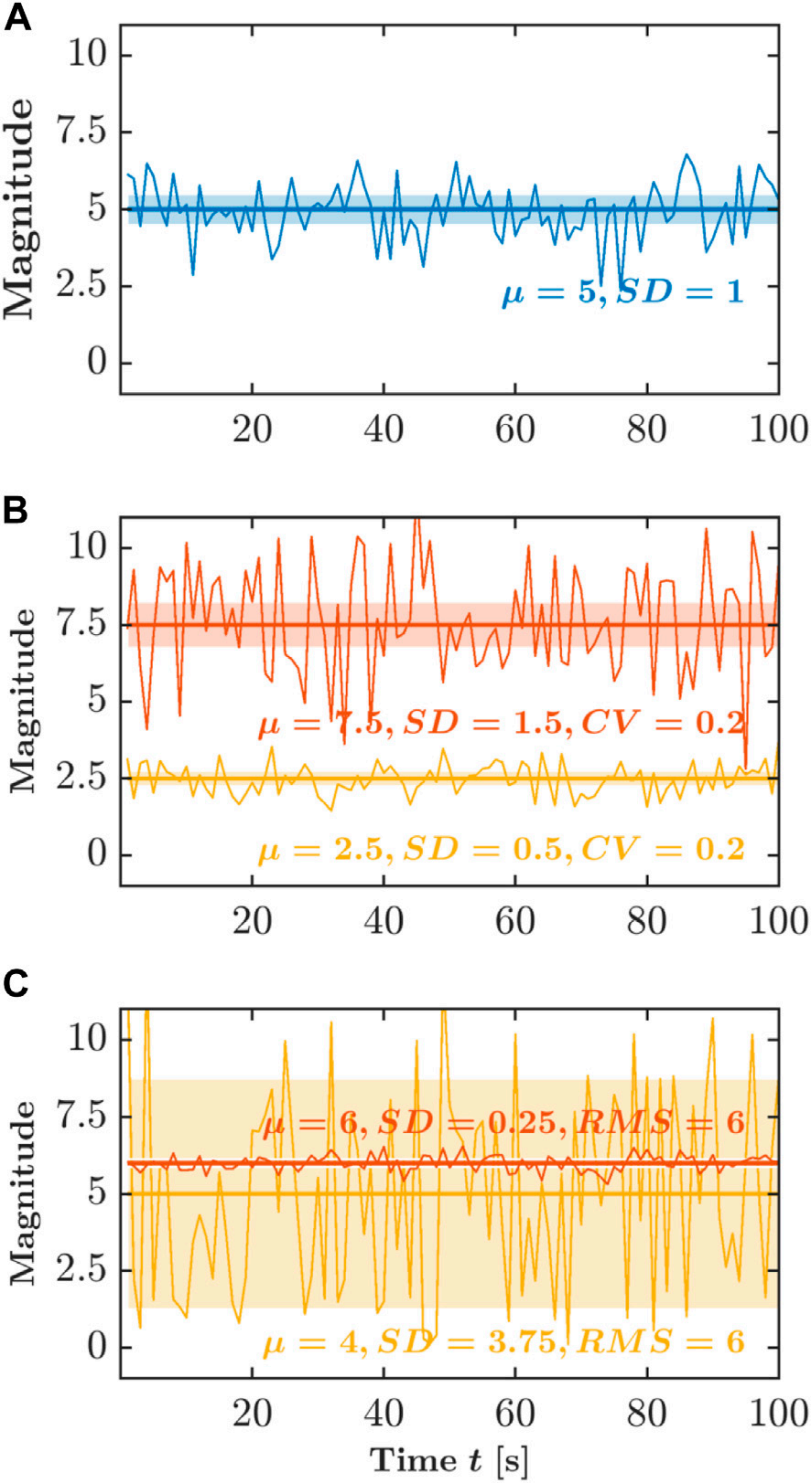
Schematic portrayal of the three typical descriptors of variability. **(A)** Standard deviation, SD. **(B)** Coefficient of variation, *CV*. **(C)** Root mean square, *RMS*. Those descriptors produce measurement summaries that emphasize their additive components—the *mean* and SD, both of which descriptors assume a summing together of very many independent random factors. *Y*-axis is shown in arbitrary units.

Human locomotion varies from one stride to the next as it adjusts to the demands of one’s environment. As the person starts walking or running, the stride interval increases to an average above zero. As the person maintains a steady pace, the average of the stride intervals holds steady, but as the foot is placed in each strike to maintain the self-selected pace, the stride interval varies from one stride to the next. The standard deviation, SD, of the stride interval time series can be used to quantify how the stride intervals vary on average (Figure 1A). Of course, the variations in stride intervals sometimes grow longer as stride intervals grow longer. The coefficient of variation, *CV*, controls for this possibility by scaling the SD relative to the central tendency, that is, SD divided by the *mean* (Figure 1B). Another way to describe the relationship between *mean* and SD is to calculate the root mean square, *RMS*. Whereas SD summarizes deviation around the *mean* and *CV* controls this summary for the size of the measurements, *RMS* expresses how much bulk variation there is in stride intervals, that is, both the central tendency’s difference from zero and the amount of variation (Figure 1C). As the person maintains a steady pace, consecutive stride intervals show progressively more variation. Indeed, upon examining the stride-to-stride variations in greater detail, the stride intervals at one point might correlate with stride intervals at another time. Stride intervals will be similar, with greater similarity likely between two strides closer in time. If we consider longer timescales, there is also more room for the stride intervals to vary. So, temporal correlations of stride intervals will decay. However, an important question is how slowly those temporal correlations decay. For instance, a gradual adjustment in foot placement to improve balance may mean that stride intervals now have long-range relationships with stride intervals much later, as the body enacts the slow change in gait.

**FIGURE 2:**
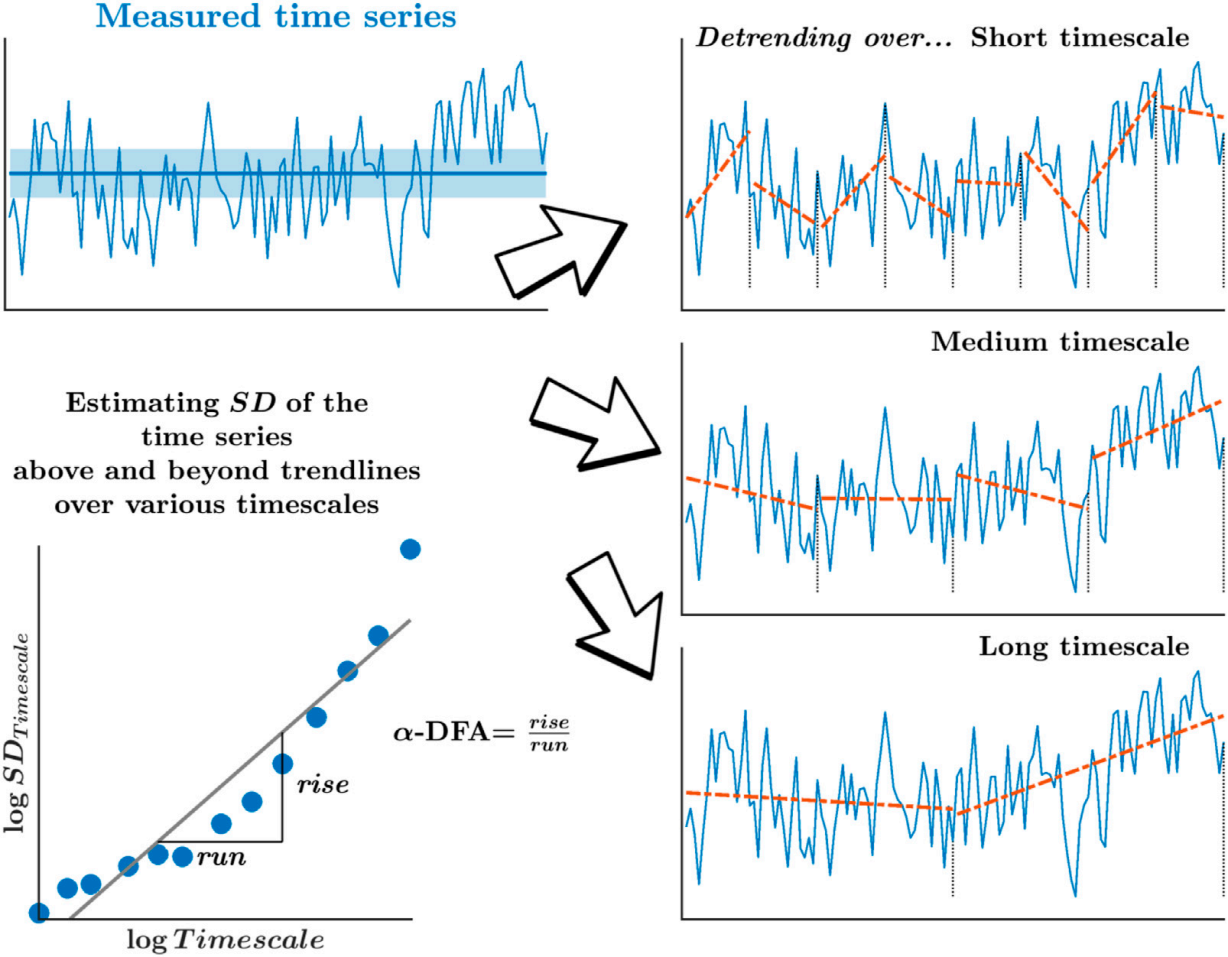
Schematic portrayal of the measure of monofractality, *α*-DFA, yielded by the detrended fluctuation analysis (DFA). *α*-DFA relates how the SD-like variation grows across many timescales, statistically encoding how the correlation among sequential measurements might decay slowly across longer separations. Detrending of those variations over progressively longer timescales removes the mean drift across each of those timescales.

Monofractality, as measured by the *α*-DFA, yielded by the so-called detrended fluctuation analysis (DFA), offers a compelling analytical method to describe how those temporal correlations between stride intervals decay across longer separations in time. Specifically, *α*-DFA relates with how the SD-like variations in stride intervals grow across many timescales, encoding how the correlation among sequential stride intervals might decay slowly across longer separations in time (Figure 2). The *α*-DFA reveals the degree of persistent correlations (0.5 < *α*-DFA < 1.0; large values in the time series are typically followed by large values and *vice versa*) or anti-persistent correlations (0 < *α*-DFA < 0.5; large values in the time series are typically followed by small values and *vice versa*) in stride-to-stride variations over time. In addition, a stride interval time series whose *α*-DFA is 0.5 has a frequency spectrum representing “white noise”—a process with equal intensity at different frequencies, giving it a constant power spectral density. In contrast, a stride interval time series whose *α*-DFA is 1 has a frequency spectrum representing “pink noise”—a process with a frequency spectrum such that the power spectral density is inversely proportional to the signal’s frequency.

Evidence of monofractality in stride-to-stride variations has been the mainstay of the optimal movement variability hypothesis (OMVH) (Harrison and Stergiou, 2015; Stergiou et al., 2006; Stergiou and Decker, 2011). In this theoretical model, unhealthful and less adaptable systems exhibit either variations that are very similar over time, resulting in a stiff, inflexible, and highly predictable behavior, or variations that are very dissimilar and random, resulting in an erratic, unfocused, and unexpected behavior (i.e., anti-persistent correlations or white noise). In contrast, healthy and highly adaptable systems display optimal variability. Monofractality in stride-to-stride variations closely describes this ideal state as it implies a temporal structure in consecutive strides that are ordered, stable but also variable, and adaptable (i.e., persistent correlations or pink noise). Many studies have also reported monofractality in stride-to-stride variations in walking (Bollens et al., 2010; Chien et al., 2015; Ducharme et al., 2018; Ducharme and van Emmerik, 2018; Fairley et al., 2010; Hausdorff et al., 1995; Hausdorff et al., 1996; Jordan et al., 2009; Terrier and Dériaz, 2011; Wilson and Likens, 2023) and running (Agresta et al., 2019; Bellenger et al., 2019; Brahms et al., 2020; Fuller et al., 2016; Fuller et al., 2017; Hoos et al., 2014; Jordan et al., 2006; Lindsay et al., 2014; Mann et al., 2015a; Mann et al., 2015b; Meardon et al., 2011; Mo and Chow, 2019; Nakayama et al., 2010; Wilson and Likens, 2023), and locomoting under various manipulations of task constraints both on treadmill (Agresta et al., 2019; Bellenger et al., 2019; Bollens et al., 2010; Chien et al., 2015; Ducharme et al., 2018; Ducharme and van Emmerik, 2018; Fairley et al., 2010; Fuller et al., 2016; Fuller et al., 2017; Hausdorff et al., 1995; Hausdorff et al., 1996; Jordan et al., 2006; Jordan et al., 2007; Jordan et al., 2009; Lindsay et al., 2014; Mann et al., 2015a; Mann et al., 2015b; Meardon et al., 2011; Mo and Chow, 2019; Nakayama et al., 2010; Terrier and Dériaz, 2011; Wilson and Likens, 2023) and overground (Bollens et al., 2010; Hoos et al., 2014; Brahms et al., 2020). However, stride-to-stride variations show a loss of monofractality in older adults (Hausdorff et al., 1997; Hausdorff et al., 2001a; Kobsar et al., 2014) and pathological populations (Hausdorff et al., 1997; Hausdorff et al., 2001a; Hausdorff, 2009). Critically, this loss of monofractality in stride-to-stride variations has been related to fall risk (Hausdorff et al., 2001b; Hausdorff, 2007; Paterson et al., 2011; Toebes et al., 2012; Johansson et al., 2016). Therefore, the predictions of the OMVH are strongly supported by the monofractality in stride-to-stride variations observed in both healthy adults and adults with a compromised movement system.

**FIGURE 3:**
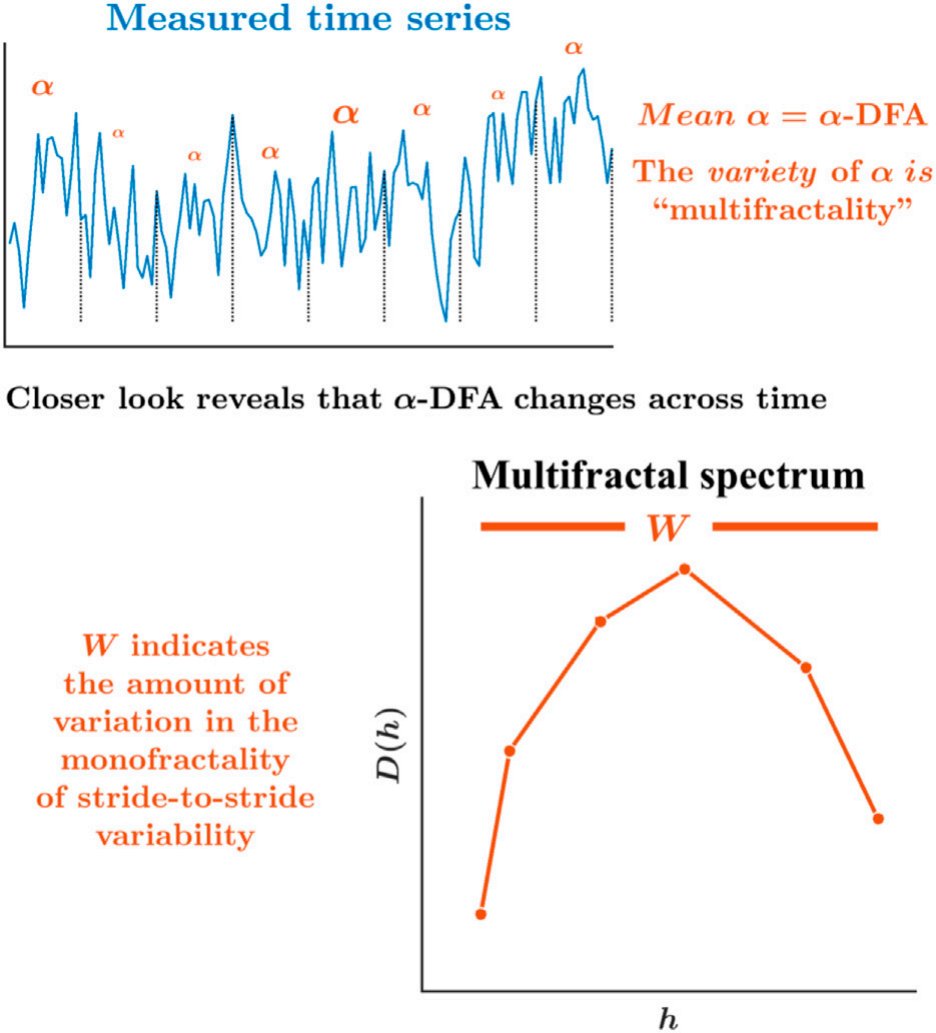
Schematic portrayal of the multifractal spectrum width, *W*, and asymmetry in the multifractal spectrum, *W*_*Asym*_. While *α*-DFA is the best single, *mean* description of the temporal structure for the whole series, peering more deeply reveals that the monofractality is changing over time. The multifractal spectrum offers the probability distribution of more local measures of monofractality across time, *α*s, and plots for each one as a frequency measure indicating how much of the time series exhibits each value of *α* (Ihlen and Vereijken, 2010; Ihlen, 2012; Ihlen and Vereijken, 2013; Kelty-Stephen et al., 2013; Kelty-Stephen et al., 2022). The relationship between the Holder/singularity exponent, *h*, and the dimension *D*(*h*) defines the multifractal spectrum. The range of *h* defines the multifractal spectrum width, *W*. In addition to estimating the heterogeneity in the fractality in terms of the multifractal spectrum width, inferences can be made from the shape of the multifractal spectrum—the rightward vs. leftward asymmetry—whether it is heterogeneity in the smaller amplitudes (or shorter stride intervals) or larger amplitudes (or longer stride intervals) that contribute to the heterogeneity of the fractality.

However, most of the above-mentioned work has focused on the monofractality of stride-to-stride variations. Addressing the possibility that the monofractality of stride-to-stride variations might vary according to time and context requires a different approach. Indeed, a growing body of work suggests that monofractality itself shows variations across experimental contexts and time with the same context (Ihlen and Vereijken, 2010; Dutta et al., 2013; Ihlen and Vereijken, 2013; Cavanaugh et al., 2017). The time-varying monofractality is termed as “multifractality.” For example, *α*-DFA might represent the dominant monofractality governing the entire stride interval time series, but there will inevitably be waxing and waning of temporal correlations around this general *α*-DFA value. Hence, constructing a “multifractal spectrum” whose width *W* indicates the diversity of temporal correlations in the same stride interval time series produces an estimation of multiple *α*-DFAs (Figure 3). Of course, human locomotion uses subtle, adaptive tunings and adjustments to ongoing changes. Expectedly, the stride-to-stride variations’ temporal structure will change over time as the participant flexibly acts to maintain a steady gait. Hence, a multifractal spectrum width gives us a more precise look at how the human locomotion system coordinates its movement across time flexibly.

In the context of gait, multifractality in stride-to-stride variations increases under faster or slower-paced walking relative to self-paced walking (Scafetta et al., 2003) and during asymmetric walking (Ducharme and van Emmerik, 2019), suggestive of more movement tuning and adjusting. Stride-to-stride variations during walking show less multifractality in individuals with neurological diseases like Parkinson’s (Dutta et al., 2013; Dutta et al., 2016) and ALS (Chatterjee, 2020), suggesting that those individuals have reduced capacity to tune and adjust movements as they move from one stride to the next. Yet, while multifractal outcomes hold significance in the context of the OMVH, the comparison between multifractal and monofractal outcomes during treadmill and overground walking and running remains uncharted territory. This gap in knowledge leaves us pondering whether the apparent monofractal behavior of stride-to-stride variations truly represents a monofractal pattern or conceals a subtler interplay between shorter and longer stride intervals influenced by the intricate web of organismic, task-related, and environmental constraints. This knowledge is important for (i) appropriately identifying the precise nature of the changes in the variability in stride-to-stride variations found in older adults and pathological populations in future experiments and (ii) implementing a more effective rehabilitation intervention based on harnessing human locomotion variability (e.g., exercises that selectively alter shorter vs. longer strides).

This study examined the effects of self-paced walking vs. running and treadmill vs. overground locomotion on standard deviation, SD, a measure of monofractality, *α*-DFA, multifractal spectrum width, *W*, and asymmetry in the multifractal spectrum, *W*_*Asym*_, of stride interval time series, to investigate whether stride-to-stride variations are similarly sensitive to various task constraints. We hypothesized that monofractality and multifractality in stride-to-stride variations would provide different insights into the ability to continually tune and adjust our movements during self-paced walking and running under various task constraints. We predicted that the greater inertia to maintain speed across gait cycles in running compared to walking would be associated with more persistent stride-to-stride variations, indicated by larger *α*-DFA, and lesser ability to tune and adjust movements, indicated by smaller *W*. We also predicted that because of the increased presence of shorter stride intervals typically found in walking than running, greater heterogeneity in shorter stride intervals than longer stride intervals would contribute to greater multifractality in walking compared to running, indicated by larger *W*_*Asym*_. However, we made no directional prediction about how monofractality and multifractality in stride-to-stride variations would differ between walking or running on the treadmill and overground. Finally, we predicted the effects of treadmill vs. overground on walking and running would be the same. Ultimately, we expected this work to illuminate the intricate dynamics of stride-to-stride variations and their responses to diverse constraints.

## 2 Methods

### 2.1 Participants

Eight adults (5 women; *mean* ± 1*s*.*d*. age: 30.5 ± 11.5 years) participated in exchange for a monetary reward. All participants (i) were able to provide informed consent; (ii) were able to walk independently without an assistive device; (ii) did not self-report any neurological disease; and (iv) did not self-report any lower limb disability, injury, or disease. Monte Carlo simulation across 5,000 iterations using the method described by Kuznetsov and Rhea. (2017) revealed that with the length of the stride interval time series of ∼800 , expected *mean α*-DFA of 0.75 and 0.98 for walking and running, respectively, inter-participant variability of 0.09 (Choi et al., 2015), trial-level variability of 0.1, and unaccounted error of 20% of true inter-subject variability, a minimum *N* = 7 participants is needed to detect a difference of 0.1 between two conditions (medium effect) at a Type I error rate of 5% with a power of >80% . Therefore, our sample size of *N* = 8 participants was expected to be enough to detect differences in the *α*-DFA between walking and running.

Each participant gave informed written consent with full knowledge of the study objectives and details of the experimental procedure. The Institutional Review Board of the University of Nebraska Medical Center approved the present study (IRB # 511-16-EP) following the Declaration of Helsinki.

### 2.2 Experimental design, procedure, and protocol

Participants performed treadmill walking (TW) and treadmill running (TR) on a Bodyguard Commercial 312C Treadmill (Priority1Fitness Inc., Launceston, England), which has a maximum speed of 12.0 mph and increases/decreases in speed by 0.1 mph. Participants also performed overground walking (OW) and overground running (OR) in the Health and Kinesiology building of the University of Nebraska at Omaha’s indoor track, which extends 200 m looping track and consists of inner, middle, and outer lanes.

Participants wore a Trigno™ 4 Contact FSR (Force Sensitive Resistor) sensor (Delsys Inc., Boston, MA) on each foot. The first and second channels registered relative pressure at the heel and midfoot, respectively, whereas the third and fourth channels were not utilized. The two FSR leads trailed around the lateral malleolus bone of each foot, placed on the heel and midfoot, and taped for security. The FSR sensor and the remaining two FSR leads were placed and strapped around the belly of the gastrocnemius muscle of the ipsilateral leg. A Trigno™ Personal Monitor (TPM) datalogger—a physiological wireless monitoring device—attached to the participant’s body stored the relative pressure data registered by the Trigno™ 4 Contact FSR sensors.

Participants performed four 20-min trials across 2 days. The first day consisted of walking and running either on the indoor track or the treadmill. The second day, separated by at least two but less than 7 days, consisted of performing on the surface that was not completed on the first day. Two familiarization trials were conducted on the treadmill day to estimate the participant’s preferred walking and running speeds based on a previously established protocol (Martin et al., 1992). The treadmill speed was increased by 0.1 mph every 2 s until the participant indicated (based on their subjective assessment) that the speed was too fast to walk/run comfortably. The treadmill speed was then reduced by 0.1 mph every 2 s until the participant indicated (based on their subjective assessment) that the speed was too slow to walk/run comfortably. Three “fast speeds” and three “slow speeds” were recorded and averaged to determine the participant’s preferred walking and running speeds. Once the preferred walking speed was estimated, the participant walked for 20 min. After 5–10 min rest, the participant’s preferred running speed was estimated, following which the participant ran at that speed for 20 min.

Heel strikes were identified based on the timestamp corresponding to the peak pressure of each foot strike from the FSRs. Stride intervals were calculated by taking the peak of *n*th heel strike of the left foot minus the peak of (*n* − 1)th heel strike of the same foot for all heel strike times in the time series. The trials yielded stride interval time series of different lengths. To keep the length of the stride interval time series constant across participants, the first 983 strides for walking and 1,527 strides for running were selected.

### 2.3 Assessing monofractality in stride-to-stride variations

Detrended fluctuation analysis (DFA), as described by Peng et al. (1994), Peng et al. (1995), assessed monofractality in stride-to-stride variations. DFA computes the exponent *α*-DFA, quantifying the strength of temporal correlations across scales using the first-order integration of time series *x*_*t*_ of length *N*, where t∈N .

$$X_{t}=\overset{N}{\underset{i=1}{\sum }}\left(x_{i}-\left\langle x\right\rangle \right),$$ (1)

where ⟨*x*⟩ is the grand mean of the time series. It computes the root mean square (*RMS*; i.e., averaging the residuals) for each linear trend *Y*_*t*_ fit to non-overlapping *n*-length bins to build fluctuation function

$$f\left(n\right)=\sqrt{\frac{1}{N}\overset{N}{\underset{t=1}{\sum }}\left(X_{t}-Y_{t}\right)},$$ (2)

for *n* < *N*/4. On standard scales, *f*(*n*) is a power law

$$f\left(n\right)∼n^{\alpha },$$ (3)

where *α* is the scaling exponent estimable using logarithmic transformation

$$\log f\left(n\right)=\alpha \log n.$$ (4)

Higher *α*, or better yet, *α*-DFA, corresponds to stronger temporal correlations. An *α*-DFA = 0.5 indicates a random time series characterized as additive white Gaussian noise (awGn), where each stride is completely uncorrelated with any previous stride. An *α*-DFA < 0.5 indicates anti-persistence in stride-to-stride variations, that is, shorter stride intervals and *vice versa* typically follow longer stride intervals. An 0.5 < *α*-DFA < 1 indicates longer stride intervals, and *vice versa* typically follows persistence in stride-to-stride variations, that is, longer stride intervals. A bin size range of [4, *N*/4] was used for the DFA in the present study, which is standard practice while using DFA (Jordan et al., 2006; Damouras et al., 2010; Farag et al., 2013; Likens et al., 2015; Likens and Stergiou, 2020).

### 2.4 Assessing multifractality in stride-to-stride variations

Multifractal wavelet leader (MFWL) analysis, as described in Wendt and Abry (2007), assessed multifractality in stride-to-stride variations. MFWL is based on the possibility that the stride interval time series could be broken down into a frequency domain using Fourier transforms to measure the changes in stride-to-stride variations (Ferree and Hwa, 2003; Ihlen and Vereijken, 2010). Fourier transforms fit sine and cosine waves to the time series data and detects correlations between the fitted waves and the time series under analysis. This fitting emphasizes the variation in shorter and longer stride intervals in the frequency domain. For instance, variation in longer stride intervals will be more apparent by fitting large amplitude sine and cosine waves to the stride interval time series. In contrast, variation in shorter stride intervals will be more apparent by fitting smaller amplitudes of sine and cosine waves to the stride interval times series. Although this analytical method must give pertinent information about the variety of shorter and larger strides, sudden changes in strides are sometimes missed by this analysis but can be detected by wavelet coefficients (Ihlen and Vereijken, 2010; Mandelbrot, 2013).

Not unlike the sine and cosine waves in Fourier transforms, wavelet coefficients detect correlations between the time series and a template waveform, which is defined as the mother wavelet, *ψ*_0_(*t*) (Wendt and Abry, 2007). The collection of mother wavelet templates characterizes the *ψ*_0_(*t*).

$$\psi_{j,k}\left(t\right)=2^{-j}\psi_{0}\left(2^{-j}t-k\right),j\in N,k\in N,$$ (5)

where *j* is the scales (milliseconds, seconds, minutes, etc.), *k* is the shift of the wavelet (e.g., one to 4 seconds, two to 5 seconds, etc.), *t* is the time at which wavelet is applied, and *N* is the time series length. The mother wavelet is fitted for each specific moment *t* of the time series at each specific scale *j*. After the mother wavelet is fitted for all scales and shifts, the wavelet coefficients are obtained such that each row of *ψ*_*j*,*k*_ is transposed and multiplied by *X*, which provides a measure of the covariation between the wavelet *ψ*_*j*,*k*_ and the vector of the time series *X*.

$$d_{X}\left(j,k\right)=\left\langle \psi_{j,k}|X\right\rangle ,$$ (6)

such that *d*_*X*_(*j*, *k*) is the coefficient that measures the correlation between the scaled mother wavelet and the original time series during each moment.

Once the wavelet coefficients have been identified, the wavelet leaders *L*_*X*_(*j*, *k*) are defined. After computing each wavelet coefficient, *d*_*X*_(*j*, *k*), the absolute value (amplitude) of each coefficient at each scale is saved. The wavelet coefficient at the scale of observation is then compared with the two wavelet coefficients at the next finer scale of observation, defined as the dyadic interval, in which the wavelet coefficient at each finer scale of observation is half the size of the coarser scale of observation. The maximum wavelet coefficient of the dyadic interval is saved for that specific dyadic interval. The observation of wavelet coefficients is then shifted (shift in *k*), and determining the maximum wavelet coefficient for each dyadic interval on that specific observation scale *j* is repeated. Finally, each maximum wavelet coefficient for each dyadic interval on that specific scale *j* is compared, in which the maximum value of all maximum wavelet coefficients is taken as the wavelet leader for that scale. Thus, there is one wavelet leader neighborhood of observation, which is defined as

$$L_{X}\left(j,k\right)=\max_{\lambda^{\prime }\in 3\lambda_{j,k}}\left\langle \psi_{j,k}|X\right\rangle ,$$ (7)

where *L*_*X*_(*j*, *k*) is a matrix of each maximum wavelet coefficient, *d*_*X*_(*j*, *k*), in the neighborhood of observation, *λ*′ ∈ 3*λ*_*j*,*k*_.

The wavelet leaders are then related to the power law

$$S^{L}\left(q,2^{j}\right)=\frac{1}{n_{j}}\overset{N_{j}}{\underset{k=1}{\sum }}L_{X}{\left(j,k\right)}^{q}=F_{q}|2^{j}{|}^{\zeta \left(q\right)},$$ (8)

where *n*_*j*_ ≈ *n*/2^*j*^ is the number of wavelet leaders at each scale *j* and *ζ*(*q*) is the spectrum of *q* exponents where small values of *q* are defined by the regular appearance of intermittent periods with little variability, and large values of *q* are defined by the regular appearance of intermittent periods with large variability. When *q* = 2, *ζ*(*q*) behaves like *α*-DFA and defines the average fractal behavior of the time series.

The Holder/singularity exponent, *h*, defined as the measure of the time series’ regularity, detects the amount and time when discontinuity occurs in a time series, and the dimension, *D*(*h*), is then calculated by the Legendre transform of *ζ*(*q*).

$$h=\frac{d\zeta \left(q\right)}{dq},$$ (9)

$$D\left(h\right)=qh-\zeta \left(q\right)$$ (10)

The relationship between *h* and *D*(*h*) defines the multifractal spectrum, and the range of *h* defines the multifractal spectrum width, *W*.

$$W=\max\left(h\right)-\min\left(h\right).$$ (11)

**FIGURE 4:**
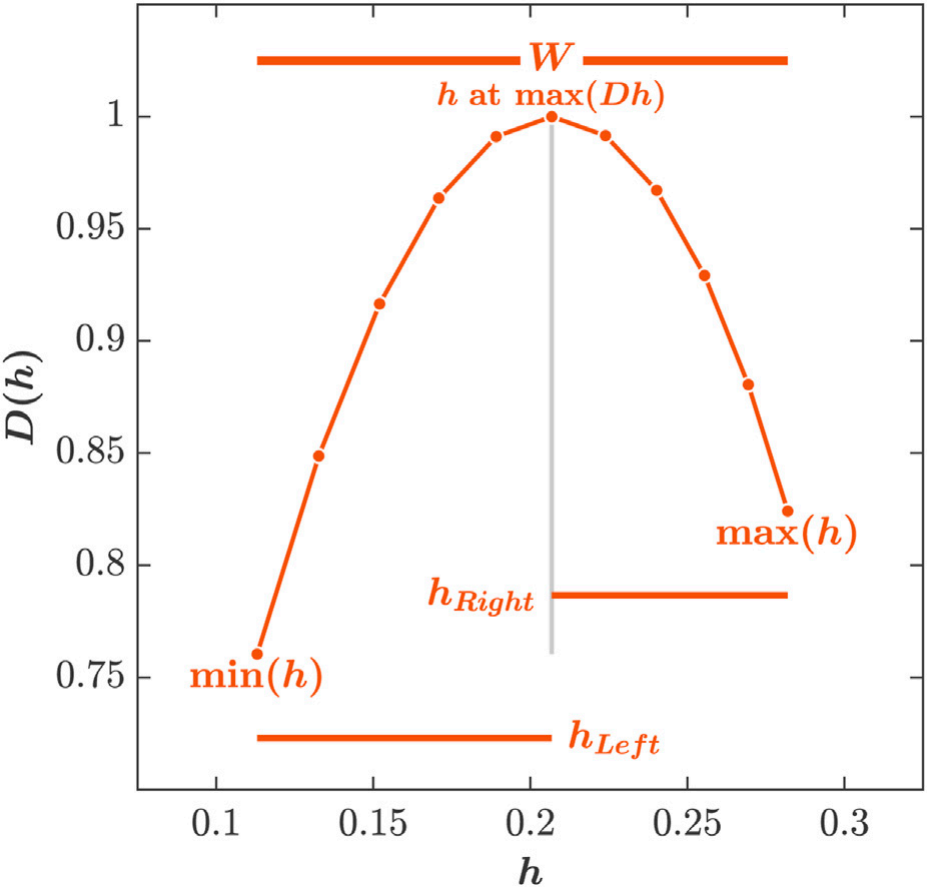
Assessing multifractal spectrum width, W, and asymmetry in the multifractal spectrum, *W*_*Asym*_ of stride-to-stride variations. Multifractal spectrum of stride interval time series was created by plotting the singularity Dimension, *D*(*h*), as a function of the Holder/singularity exponent, *h*. The multifractal spectrum width is determined as *W* = max(*h*) − min(*h*). Variation in shorter stride intervals was determined as *h*_*Right*_ = max(*h*) − *h* at max(*Dh*), whereas variation in longer stride intervals was determined as *h*_*left*_ = *h* at max(*Dh*) − max(*h*). *W*_*Asym*_ was determined as *W*_*Asym*_ = *h*_*Left*_ − *h*_*Right*_, with a positive *W*_*Asym*_ indicative of more variation in longer stride intervals, while a negative *W*_*Asym*_ indicative of more variation in shorter stride intervals.

To determine asymmetry in the multifractal spectrum, the multifractal spectrum is separated into two lateral parts by the maximum magnitude, *h* at max(*Dh*). Variation in the structure of shorter stride intervals is defined by max(*h*) − *h* at max(*Dh*), denoted as *h*_*Right*_. Variation in the structure of longer stride intervals is defined by *h* at max(*Dh*) − min(*h*), denoted by *h*_*Left*_. Asymmetry in the multifractal spectrum, *W*_*Asym*_, reflecting the variation of the structure between shorter and longer stride intervals, is defined as

$$W_{\mathrm{Asym}}=h_{\mathrm{Left}}-h_{\mathrm{Right}},$$ (12)

where a positive *W*_*Asym*_ is indicative of more variation in longer stride intervals, while a negative *W*_*Asym*_ is indicative of more variation in shorter stride intervals (Figure 4).

### 2.5 Statistical analysis

Although commonly used in most scientific literature, the dichotomous interpretation of the frequentist *p*-value has been criticized in recent years for theoretical, practical, and ethical reasons (Dixon, 2003; Cumming, 2008; Wetzels et al., 2011; Cohen, 2016; Wagenmakers et al., 2018b). Therefore, a Bayesian analytical approach was used, which focuses on weighing evidence in favor of both the null and alternative hypotheses. Four two-way Bayesian repeated measure ANOVAs with default priors (Wagenmakers et al., 2018a; Wagenmakers et al., 2018b) were performed to investigate the effects of locomotion mode (walking vs. running) and surface (treadmill vs. overground) on the temporal structure of stride-to-stride variations: standard deviation, SD, the measure of monofractality, *α*-DFA, estimated using the DFA, the multifractal spectrum width, *W*, estimated using MFWL analysis, and the multifractal spectrum width asymmetry, *W*_*Asym*_, estimated using MFWL analysis. In the present study, objective Bayesian ANOVAs with default Cauchy priors were implemented.

As one alternative to the *p*-value in the frequentist statistics, the Bayesian approach employs and interprets the Bayes Factor (*BF*_10_), defined as the ratio of the information in favor of the alternative hypothesis compared to the null hypothesis or *vice versa*. For example, a *BF*_10_ = 2 means the alternative hypothesis is two times more likely than the null hypothesis. To interpret the proceeding results, the following *BF*_10_ intervals are defined as the strength of evidence in support of the alternative hypothesis: 1 < *BF*_10_ < 3 represents anecdotal (i.e., weak or limited) evidence, 3 ≤ *BF*_10_ < 10 represents substantial evidence, 10 ≤ *BF*_10_ < 30 represents strong evidence, 30 ≤ *BF*_10_ < 100 represents very strong evidence and 100 ≤ *BF*_10_ represents decisive evidence. *BF*_10_ = 1 indicates no evidence in favor of either the null or alternative hypothesis. Alternatively, the following *BF*_10_ intervals are defined as the strength of evidence in support of the null hypothesis: 1/3 ≤ *BF*_10_ < 1 represents anecdotal evidence, 1/10 ≤ *BF*_10_ < 1/3 represents substantial evidence, 1/30 ≤ *BF*_10_ < 1/10 represents strong evidence, 1/100 ≤ *BF*_10_ < 1/30 represents very strong evidence, and *BF*_10_ < 1/100 represents decisive evidence (Wetzels et al., 2011). An important qualifier is that interpretive ranges are not meant to be absolute boundaries. Treating *BF*_10_ as such reintroduces many of the same problems associated with *p*-values. Post hoc tests investigated between-trial differences in the SD, *α*-DFA, *W*, and *W*_*Asym*_. Posterior odds were corrected for multiple comparisons (Westfall et al., 1997). All statistical analyses were performed in JASP 0.18.1 (Love et al., 2019).

## 3 Results

**FIGURE 5:**
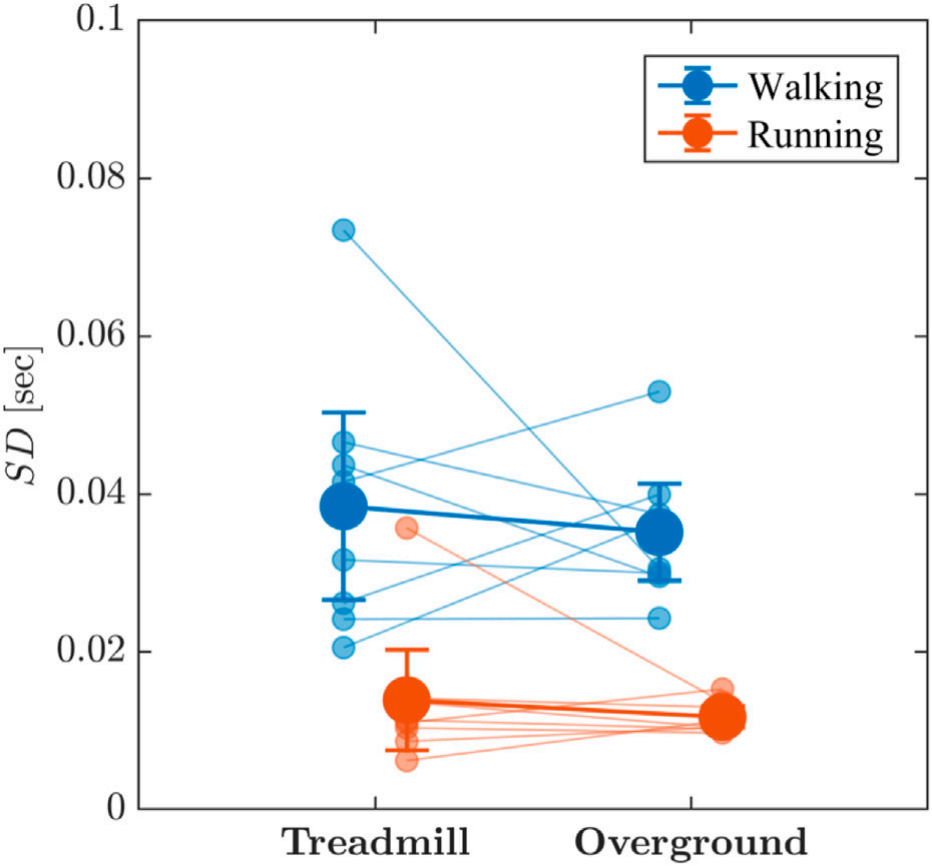
Line graph depicting the effects of locomotion mode (walking vs. running) and surface (treadmill vs. overground) on the standard deviation, SD, of stride-to-stride variations. Light blue and light red circles indicate SD values for individual participants in the respective conditions. Vertical bars indicate 95% Credible Intervals (*N* =8 participants).

Visual inspection indicated that the standard deviation, SD, of stride-to-stride variations were greater for walking than for running and for treadmill locomotion than for overground locomotion (Figure 5). There was very strong evidence that walking produced greater SD of stride-to-stride variations than did running (*BF*_10_ = 69.515) and limited evidence that treadmill and overground SD were, on average, nearly equivalent (*BF*_10_ = 0.491). There was also anecdotal evidence suggesting no additional locomotion mode × surface interaction effect concerning influencing SD (*BF*_10_ = 0.581).

**FIGURE 6:**
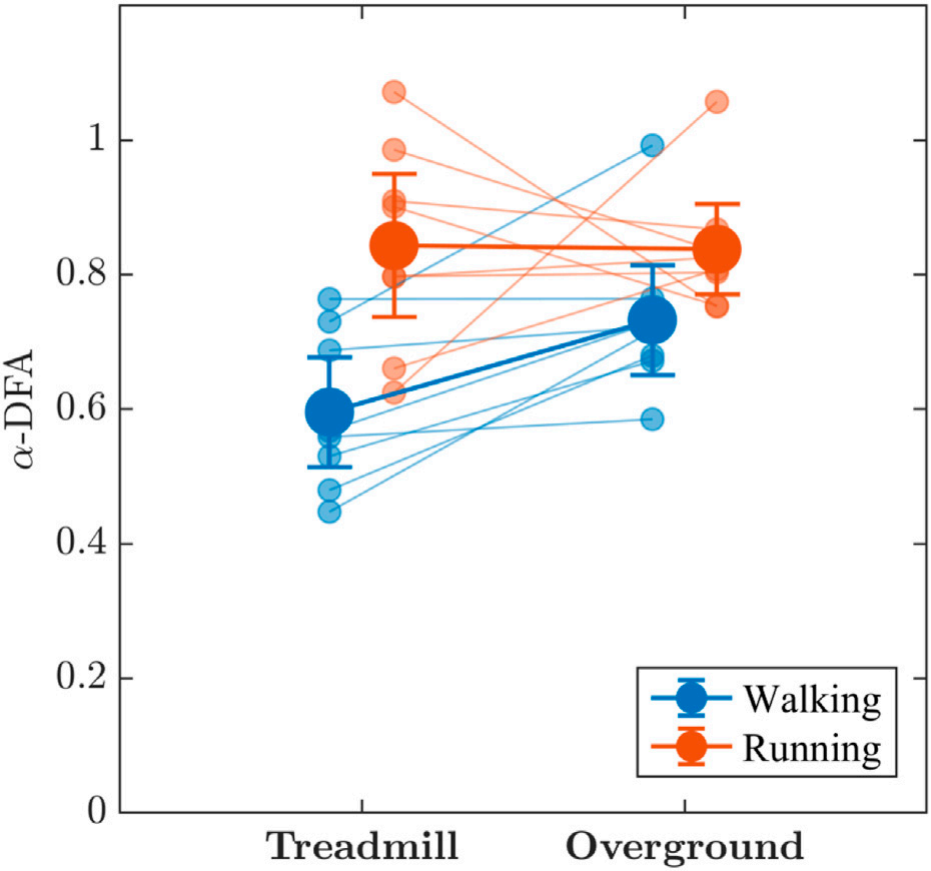
The effects of locomotion mode (walking vs. running) and surface (treadmill vs. overground) on the measure of monofractality, *α*-DFA, in stride-to-stride variations. Light blue and light red circles indicate *α*-DFA values for individual participants in the respective conditions. Vertical bars indicate 95% Credible Intervals (*N* =8 participants).

The values of the measure of monofractality, *α*-DFA, of stride-to-stride variations were consistently larger than 0.50 across walking and running, even approaching 1 for a few participants (Figure 6), indicating strong persistence in stride-to-stride variations, both on the treadmill and overground surface. There was substantial evidence that *α*-DFA was higher when running compared to walking (*BF*_10_ = 8.976) and weak evidence that overground locomotion produced higher *α*-DFA (*BF*_10_ = 1.500) than treadmill locomotion; however, those effects appear to be modified by an interaction *α*-DFA (*BF*_10_ = 3.206) such that differences in *α*-DFA between walking and running are most evident during treadmill locomotion where *α*-DFA when walking (*BF*_10_ = 5.22) appears lower than when running (*BF*_10_ = 3.28).

**FIGURE 7:**
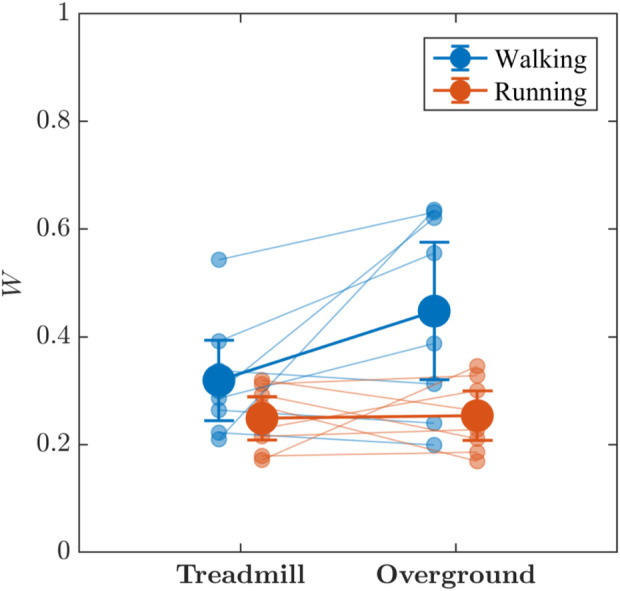
The effects of locomotion mode (walking vs. running) and surface (treadmill vs. overground) on the multifractal spectrum width, *W*, of stride-to-stride variations. Light blue and light red circles indicate *W* values for individual participants in the respective conditions. Vertical bars indicate 95% Credible Intervals (*N* = 8 participants).

Visual inspection of Figure 7 suggests the locomotion mode and surface may jointly influence the multifractal spectral width, *W*, of stride-to-stride variations, which was greater for walking than for running, with weak evidence supporting the presence of an interaction effect, (*BF*_10_ = 2.428). Additionally, substantial evidence indicated that walking produced greater *W* of stride-to-stride variations (*BF*_10_ = 3.815) than running and that overground locomotion produced greater *W* (*BF*_10_ = 1.359) than did treadmill locomotion; however, due to the interaction, no further interpretation is needed. Post hoc tests revealed that as expected from Figure 7, there was substantial evidence that overground walking produced greater *W* than did overground running (*BF*_10_ = 3.16). In contrast, there was weak evidence that treadmill running and walking produced comparable *W* (*BF*_10_ = 0.72).

**FIGURE 8:**
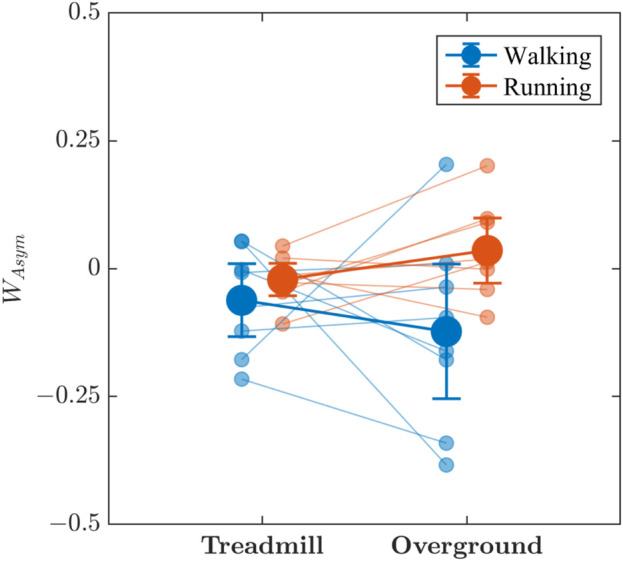
The effects of locomotion mode (walking vs. running) and surface (treadmill vs. overground) on the asymmetry in the multifractal spectrum width, *W*_*Asym*_, of stride-to-stride variations. Light blue and light red circles indicate *W*_*Asym*_ values for individual participants in the respective conditions. Vertical bars indicate 95% Credible Intervals (*N* =8 participants).

Visual interpretation of Figure 8 is less clear than those depicting other measures of stride-to-stride variations. Asymmetry in the multifractal spectrum width, *W*_*Asym*_, of stride-to-stride variations was mostly negative for walking (i.e., rightward skew in the multifractal spectrum) and close to zero for running, and there is some indication that walking produced greater *W*_*Asym*_ than did running. That trend implies more variability in the fractal structure involving large fluctuations in stride intervals. In line with the visual inspection, there is weak evidence that *W*_*Asym*_ was greater while running than when walking (*BF*_10_ = 1.458), as well as weak evidence that treadmill and overground locomotion produced similar *W*_*Asym*_ (*BF*_10_ = 0.518). The evidence regarding the combined effect of locomotion mode and surface on *W*_*Asym*_ was inconclusive (*BF*_10_ = 0.936).

## 4 Discussion

To investigate whether stride-to-stride variations are similarly sensitive to various task constraints, we examined the effects of self-paced walking vs. running and treadmill vs. overground locomotion on standard deviation, SD, a measure of monofractality, *α*-DFA, multifractal spectrum width, *W*, and asymmetry in the multifractal spectrum, *W*_*Asym*_, of stride interval time series. The results supported our hypothesis that monofractality and multifractality in stride-to-stride variations provide different insights into the ability to continually tune and adjust our movements during self-paced walking and running. For instance, we found greater *α*-DFA values for running than walking, possibly due to the greater inertia in running. In contrast, we found greater *W* values for walking than running, suggesting the greater ability to tune and adjust movements during walking than during running. This latter finding was strongly supported by the large SD in stride-to-stride variations for walking compared to running. We also found a more leftward skew in the multifractal spectrum encoded by more negative *W*_*Asym*_ for walking than running, indicative of more variability in the fractal structure involving smaller fluctuations in stride intervals. The multifractal spectrum for running was mostly symmetric, most likely due to the relative absence of shorter stride intervals.

The effects of the locomotion mode were observed to have an interesting interaction with the locomotion surface. Specifically, our study found that walking on a treadmill resulted in smaller *α*-DFA values than walking overground, whereas walking overground was associated with larger *W* values than walking on a treadmill. Essentially, walking overground demonstrated greater persistence in stride-to-stride variations, but this persistence also exhibited more pronounced waxing and waning. This contrasts with the locomotion mode, where lesser persistence during walking waxed and waned, while greater persistence during running was relatively stable. Hence, the interconnectivity between persistence and waxing/waning tendencies for the locomotion surface underscores a more nuanced perspective on how diverse task constraints can affect various statistical aspects of stride-to-stride variations, providing a window into the tuning and adjustments of movements.

Our study revealed that walking is noisier than running in terms of stride-to-stride variations and has a higher standard deviation. Specifically, we found that walking resembles white noise, while running is closer to pink noise, indicating stronger temporal correlations. Additionally, the multifractal spectrum, which measures the variety in the fractality of the stride-to-stride variations, supports this trend by showing greater variety in fractality during walking but less during running. These findings raise the question of why stronger temporal correlations in running are associated with less variety in those correlations, while weaker temporal correlations in walking are associated with more variety. Our proposed explanation is that running allows less time for consecutive strides, which limits the ability to make adjustments compared to walking. This time constraint reduces the variability of temporal correlations during running while allowing for a greater ability to tune and adjust movements during walking. Further research can be conducted to test this hypothesis. Overall, our study sheds light on the differences in stride-to-stride variability between walking and running and provides a basis for exploring the underlying mechanisms of these variations.

Critically, embedding the single stride in the multiscaled structure encoded by the multifractal spectrum provides an interesting perspective regarding interpretation based on the optimal movement variability hypothesis. The optimal movement variability hypothesis posits that healthy adults exhibit stride-to-stride variations that balance stability and flexibility, enabling them to adapt to the demands of the task and environment (Stergiou et al., 2006; Stergiou and Decker, 2011; Harrison and Stergiou, 2015). Accordingly, healthy individuals persist in stride-to-stride variations, whereby shorter strides tend to follow shorter strides, and longer strides tend to follow longer ones. Previous research has consistently reported such persistence across walking and running tasks (Hausdorff et al., 1996; Hausdorff et al., 2001a; Jordan et al., 2006; Bollens et al., 2010; Fairley et al., 2010; Terrier and Dériaz, 2011; Lindsay et al., 2014; Mo and Chow, 2019). Similarly, we observed an 0.5 < *α*-DFA < 1.0, indicative of persistence in stride-to-stride variations during self-paced walking and running on the treadmill and overground surfaces. However, our study reveals that persistence is not a static trait but a variable subject to additional constraints (walking produced a wider multifractal spectrum, i.e., greater *W*, of stride-to-stride variations than running, and overground locomotion produced a wider multifractal spectrum, i.e., greater *W*, than treadmill locomotion). Specifically, stride-to-stride patterns can demonstrate less persistence, which may be transient (e.g., smaller *α*-DFA but larger *W* for running). Conversely, stride-to-stride variations can exhibit greater persistence, but the persistence may be more or less variable (e.g., greater *α*-DFA but smaller *W* for running). Therefore, these findings add a more detailed and nuanced understanding of movement patterns to monofractal formalism. In contrast to monofractal formalism, which provides a reference range to distinguish between healthy and unhealthy variability, multifractal formalisms offer measures of variability that provide a more detailed and nuanced understanding of movement patterns. Therefore, the present study suggests that multifractal formalisms may enable a more comprehensive analysis of the underlying processes governing movement variability, which could have significant implications for clinical applications, athletic performance, and theoretical arguments about optimal forms of movement.

However, it is important to acknowledge several limitations in our study. Firstly, the overground walking and running trials were conducted on a looping track with constant and regular turns, in contrast to the straight-line locomotion on the treadmill. At the same time, one might argue that turning requires more intricate movement tuning and adjustments; we did not observe significantly larger asymmetry in the multifractal spectrum for overground locomotion than treadmill locomotion. Therefore, this factor is unlikely to influence our results and their interpretations substantially. Secondly, our study has a narrow focus on stride-to-stride variations. A more comprehensive understanding of the specific movement adjustments made during running, as compared to walking, as we have suggested, would benefit from a deeper analysis of full-body kinematics and the intricate musculoskeletal adjustments required to accommodate the stride-to-stride adaptations. In conclusion, while our study has certain limitations, particularly regarding the track layout and the exclusive emphasis on stride-to-stride variations, these findings provide valuable insights into the multifaceted dynamics of locomotion.

The clinical implications of our findings are noteworthy. Aging and pathological populations have been shown to exhibit a loss of monofractality in stride-to-stride variations (Hausdorff et al., 1997; Hausdorff et al., 2001a; Kobsar et al., 2014), which is closely associated with increased fall risk (Hausdorff et al., 2001b; Hausdorff, 2007; Paterson et al., 2011; Toebes et al., 2012; Johansson et al., 2016). This phenomenon has been well-documented in older adults and individuals with neurological diseases such as Parkinson’s. Furthermore, individuals with neurological diseases, particularly Parkinson’s, have demonstrated diminished multifractality in their stride-to-stride variations during walking (Dutta et al., 2013; Dutta et al., 2016). The multifractal spectral characteristics of stride-to-stride variations in walking and running provide a valuable means of assessing an individual’s capacity for movement tuning and adjustments. Consequently, these descriptors have the potential to serve as quantitative tools for evaluating the effectiveness of rehabilitative interventions aimed at restoring natural stride-to-stride variations in older adults and clinical populations with gait disorders. Our results offer a promising avenue for clinicians and researchers to refine their approaches and track improvements in gait dynamics, ultimately enhancing the quality of care and intervention strategies for individuals at risk of falls or affected by neurological conditions.

The present study is the first to compare monofractal and multifractal measures of stride-to-stride variations between walking and running on the treadmill and overground surfaces. Therefore, no published results contrast the present findings. While the current results indicate that self-imposed constraints (walking vs. running) critically influence the temporal structure of stride-to-stride variations in terms of its multifractality, the effects of externally imposed constraints are only subtly apparent (treadmill vs. overground). Future research could include multiple overground environments to determine differences or lack thereof, compared to a treadmill environment to understand better how externally imposed constraints on the multifractality in stride-to-stride variations and what it entails in terms of balance for individuals with different life histories (e.g., young vs. old, healthy vs. diseased). The outcomes of the present and future studies will help identify the precise nature of the loss or change of variability in stride-to-stride variations in older adults and pathological populations in future experiments and to implement a more effective rehabilitation intervention based on the principles of the optimal movement variability hypothesis (e.g., exercises that selectively alter the statistics of shorter vs. longer strides).

## Data Availability

The original contributions presented in the study are included in the article/Supplementary Material, further inquiries can be directed to the corresponding author.
